# Is there any difference between oral preemptive pregabalin vs. placebo administration on response to EBUS-TBNA under sedation?

**DOI:** 10.3906/sag-2005-305

**Published:** 2021-02-26

**Authors:** Semih AYDEMİR, Ali ALAGÖZ, Fatma ULUS, Mehtap TUNÇ, Hilal SAZAK, Nilgün Yılmaz DEMİRCİ

**Affiliations:** 1 Department of Anesthesiology and Reanimation, Faculty of Medicine, University of Health Sciences, Atatürk Chest Diseases and Thoracic Surgery Training and Research Hospital, Ankara Turkey; 2 Department of Chest Diseases, Faculty of Medicine, Gazi University, Ankara Turkey

**Keywords:** Pregabalin, endobronchial ultrasonography, sedation, ketamine, propofol, anxiety

## Abstract

**Background/aim:**

The aim of this study is to evaluate the effects of preemptive oral pregabalin on hemodynamic response, anxiety, sedation, and recovery in patients who underwent endobronchial ultrasound-guided transbronchial needle aspiration (EBUS-TBNA) under sedation with intravenous ketamine-propofol combination.

**Materials and methods:**

Sixty patients were included in this study, and patients were randomly divided into two equal groups to receive the placebo (Group 1) versus pregabalin 150 mg (Group 2) one hour prior to EBUS- TBNA procedure. Patients received 0.25 mg kg^-1^ ketamine and 0.25 mg kg^-1^ propofol mixture (ketofol) for sedation. Timing of the parameters was defined as follows; T0: in hospital ward before pregabalin or placebo administration, T1: premedication, T2: in operating room, T3: before the procedure, T4: initiation, T5: 3 min after induction, T6: 6 min after induction, T7: 9 min after induction, and T8: 12 min after induction. Hemodynamic parameters, severity of coughing, sedation and anxiety scores, and complications were recorded. The level of satisfaction of the bronchoscopist and the patients were evaluated at the end of the procedure.

**Results:**

The heart rate and mean arterial pressure were significantly higher in Group 1 (P = 0.008, P = 0.04). Total doses of anesthetics, recovery time, and desaturation rate were significantly higher in Group 1 (P = 0.014, P = 0.001, P = 0.045). In Group 2, SpO_2_ level was significantly higher at various time periods (T1; P = 0.025, T4; P =0.043, T6; P = 0.001, T7; P = 0.003, T8; P < 0.001). The severity of coughing was found significantly lower in Group 2 (T4; P = 0.011, T5; P = 0.01, T6; P = 0.02, T7; P = 0.03, T8; P < 0.01). Anxiety scores were significantly lower in Group 2 (P < 0.001).

**Conclusion:**

Preemptive oral pregabalin, in addition to sedation with ketamine-propofol combination, was effective in providing limited hemodynamic response, restricted coughing reflex, and lower anxiety during EBUS-TBNA. Besides, with pregabalin usage, decreased anesthetics consumption, lower complication rate, and shorter recovery time might have contributed to safety of the procedure and comfort of the bronchoscopist.

## 1. Introduction

Endobronchial ultrasonography (EBUS) is a method to diagnose the structures that are close to the trachea-bronchial system. It is commonly used to diagnose the mediastinal and/or hilar lymph nodes and to guide the transtracheal and transbronchial needle aspiration (TBNA) for staging of lung cancer [1]. 

EBUS-TBNA is one of the procedures that cause high level of anxiety in patients, and it might cause the deterioration of hemodynamic parameters. It also affects the patients’ and bronchoscopists’ comfort in negative way either [2]. Sedation is essential for EBUS-TBNA to reduce anxiety while protecting the airway reflexes. In addition, a comfortable and safe condition is important for patients and bronchoscopist [2]. 

Although various sedative agents have been employed for EBUS-TBNA, any ideal agents or protocols have not yet been determined for sedation [2,3]. Different disturbing conditions might appear due to high level anxiety. Increasing anxiety and fear might cause high pain scores and consumption of high anesthetic agents. It also increases the induction dose of anesthetics [4]. 

Pregabalin is an anxiolytic agent, and it is well absorbed and tolerated using orally. Its’ peak plasma concentration is provided one hour after orally taken. Various studies have been performed to evaluate the pregabalin effect on intubation response, postoperative pain, and analgesic consumption [4-9]. Even if pregabalin was used for fiberoptic bronchoscopy with different sedative agent, there is no article related to the effects of pregabalin during EBUS-TBNA procedure with ketofol sedation.

In this study, we aimed to assess the effects of preemptive oral pregabalin on hemodynamic response, anxiety, sedation, and recovery in patients who underwent EBUS-TBNA under sedation with ketamine-propofol combination.

## 2. Materials and methods

**Figure 1:**
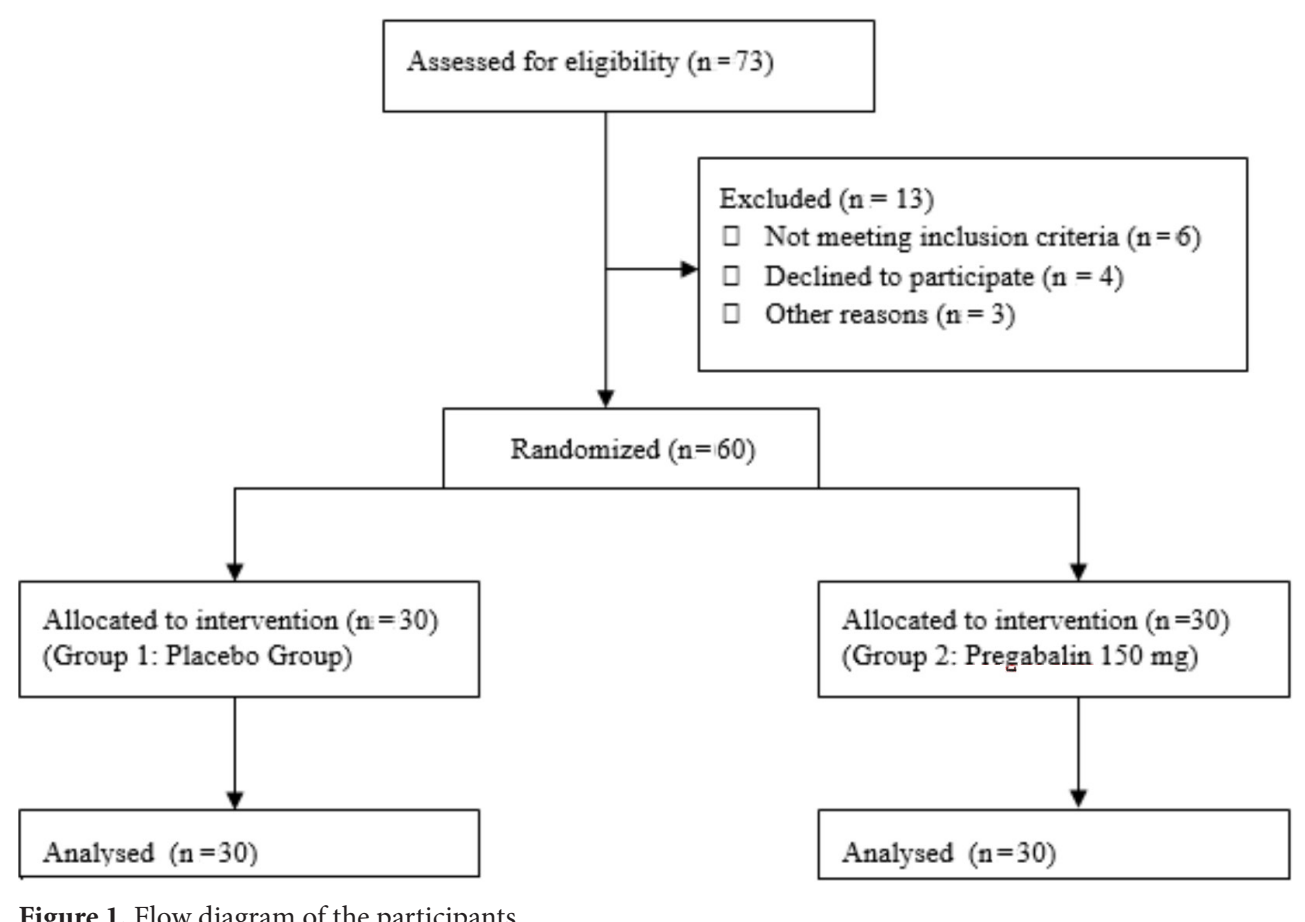
Flow diagram of the participants.

This prospective study was performed after institutional review board approval (14/08/2013, ID: 366). Sixty patients aged between 18 and 60 years, undergoing EBUS-TBNA with American Society of Anesthesiologists (ASA) physical status of I-III were included in this study. Written informed consent was obtained from all patients the day before the procedure. Exclusion criteria included history of allergy to any anesthetic agents, peripheral oxygen saturation (SpO_2_) lower than 90%, and clinical evidence of hypertension, cardiac diseases, arrhythmia, renal disease, liver failure, depression, dementia, deterioration of mental status, upper airway infection, acute asthma attack, thyroid disease, alcohol or illicit drug abuse, and electrolyte disturbance. Patients were randomly allocated into two groups with the assistance of a computer-generated table of random numbers (Figure 1). Placebo and pregabalin 150 mg were provided by hospital pharmacy, and both medications prepared as identical. The placebo capsules (Group 1, n = 30) and pregabalin150 mg (Group 2, n = 30) were given to the patients randomly one hour before the procedure orally with sips of water.

Demographic data, body mass index (BMI), ASA physical status, gender, heart rate (HR), systolic arterial pressure (SAP), diastolic arterial pressure (DAP), mean arterial pressure (MAP), SpO_2_, and respiratory rate (RR) were recorded. Ramsay sedation score (RSS), severity of coughing, and visual analog scale for anxiety (VAS-A) were also recorded. The SAP, DAP, MAP, HR, SpO_2_, RSS, RR, VAS-A, and severity of coughing were also recorded every 3 min during procedure. 0.01 mg kg^-1^ atropine and 0.03 mg kg^-1^ midazolam were administered intramuscularly for premedication. Five minutes before the procedure had started, 2% lidocaine spray was pumped ten times (1 pump = 10 mg lidocaine) into the pharynx. In the operating room, all patients received 0.03 mg kg^-1^ bolus doses of midazolam and ketofol (mixture of 1:1 ratio ketamine- propofol) intravenously for sedation. Ketofol was prepared by combining ketamine 1 mL (50 mg mL-1), propofol 5 mL (10 mg mL-1), and saline 4 mL in a single syringe. 1 mL of ketofol includes ketamine 5 mg and propofol 5 mg. The bolus dose of ketofol was titrated as 0.25 mg kg^-1^ propofol and 0.25 mg kg^-1^ ketamine for induction. 4 L min of O_2_ was administered to the patients by nasal cannula, and the flow of O_2_ was increased to 6 L min in case of SpO_2_ lower than 90%. 

The EBUS-TBNA procedure was started when the RSS was 4. The RSS was defined as 1: patient anxious, agitated; 2: patient cooperative, orientated; 3: patient responds to only verbal stimulation; 4: patient asleep, rapid response to light stimulation or loud auditory stimulus; 5: patient asleep, slow response to light stimulation or loud auditory stimulus; 6: no response to any stimulation. 

A convex probe EBUS (BF-UC 180F, Olympus Corp., and Tokyo, Japan) was used to examine the lymph nodes, and the ultrasound images were processed with a dedicated scanner (EU-ME1, Olympus Corp.). The bronchoscopist used 22-gauge needle to sample the lymph nodes and applied 2% lidocain while the bronchoscope was passing through vocal cords, carina, and bronchus. Total topical lidocaine dose was limited to 200 mg. Amounts of induction dose were recorded. The maintenance of sedation was provided with the intermittent bolus of 0.25 mg kg^-1^ propofol and 0.25 mg kg^-1^ ketamine, and the number of additional doses needed was recorded for each patient. 

Timing of the parameters was defined as follows; T0: in hospital ward before pregabalin 150 mg or placebo administration, T1: in the premedication room, T2: in operating room, T3: before the EBUS-TBNA procedure, T4: initiation of EBUS-TBNA, T5: 3 min after induction, T6: 6 min after induction, T7: 9 min after induction, and T8: 12 min after induction. 

Anxiety level of patients was evaluated by using VAS-A [10]. Anxiety level determined in chart from 0: not anxious at all to 100: extremely anxious [10]. The severity of coughing was determined as the following criteria; Grade 0: no incidence of coughing, Grade 1: only one cough, Grade 2: from mild cough to two coughs, Grade 3: severe or repetitive coughing [11].

Initial coughing value was accepted as at the beginning of EBUS-TBNA after anesthesia induction, and coughing score was recorded every 3 min. Any changing in MAP (20% increasing or decreasing) and HR (< 50 or > 120 beat/min) was accepted as blood pressure and HR alteration, respectively. Desaturation was determined as any SpO_2_ level below the 90% longer than 30 s. All hemodynamic alterations and desaturation were considered sedation related complications, and they were recorded during procedure. We also recorded bronchospasm, laryngospasm, bleeding, and pneumothorax as procedure related complications. 

The recovery time was determined by using modified aldrete score (MAS) that includes assessment of patient’s consciousness, activity, respiration, blood pressure, and oxygen saturation to determine recovery. MAS of 0-2 is given for each of the five categories, for a maximum score of 10. The total score ≥ 8 after discontinuation anesthetic agents were accepted as recovery time. After the EBUS procedure, the questionnaire was filled in by the bronchoscopist to assess the operator satisfaction. This evaluation was to answer the following questions; 1. The anesthetic method was not satisfactory, 2. The anesthetic method was moderately satisfactory, 3. Good anesthetic condition, 4. Excellent anesthetic condition. 

Another questionnaire was performed for patients to evaluate the patients’ satisfaction after 2 hours from the EBUS-TBNA. Assessment of amnesia during the procedure was performed with the following possible answers; 1. I didn’t remember anything about the procedure, 2. I remember only some parts of the procedure, 3. I remember all of the procedure clearly.

Patients also reported their comfort level with the procedure as follows; 1. I did not feel any difficulty, 2. I felt a little uncomfortable, but it was good, 3. The procedure bothered me, but it was tolerable, 4. The procedure was unbearable.

Patients were asked the following questions to assess whether they would allow for the same procedure in the future; 1. Yes, I will, 2. If EBUS is mandatory and the bronchoscopist insists on the application of this procedure, 3. No, I will not.

### 2.1. Statistical analysis

Sample size was calculated to use MAP and HR and a 15% difference in groups with 90% power and 5% error to test the statistical importance by using G-Power for Mac OS X (Universitat Düsseldorf, version 3.1). To account for potential protocol violations, the researchers included an additional two patients in each group. Mean ± standard deviation and median [min-max] were used for continuous numerical variables. Qualitative variables were summarized with numbers and percentages. A Shapiro-Wilk test was used to assess the normal distribution of all parameters related to the patients. In the case of providing parametric test assumptions between the groups in terms of numerical variables, it was examined by t test in independent groups. If these assumptions were not met, Mann-Whitney U test was used. Whether there was any difference between the groups in terms of categorical variables was examined with the Chi-square test. In terms of blood pressure, HR, and SPO_2_ changes, the difference between the groups and within the groups was examined by variance analysis in repeated measures. In case of difference, Bonferroni correction was used for paired comparisons. Within-group changes of VAS and sedation score were analyzed using Friedman test. P value < 0.05 was considered as statistically significant.

## 3. Results 

There was no statistically significant difference between the groups in terms of sex, age, ASA physical status, and BMI (P = 0.770, P = 0.535, P = 0.495, P = 0.418) (Table 1). 

**Table 1:** Demographic characteristics and American Society of Anesthesiologist physical status of patients.

	Group 1 (n = 30)	Group 2 (n = 30)	P
Sex(Male/Female)	23/7(76.7%/23.3%)	21/9(70%/30%)	0.770
ASA (I/II/III)	2/15/13(6.7%/50%/43.3%/0%)	2/18/10(6.7%/60%/33.3%)	0.495
Age/year(t)	48.1 ± 9.8	49.9 ± 11.6	0.535
BMI/kg/m2(u)	25.1 ± 3.0	25.9 ± 4.5	0.418

*P < 0.05, (t): t-test, (u): Mann-Whitney U test.ASA: American Society of Anesthesiologist. BMI: Body mass index. Age and BMI presented as mean±standard deviation. Sex and ASA presented as percentage.

**Figure 2:**
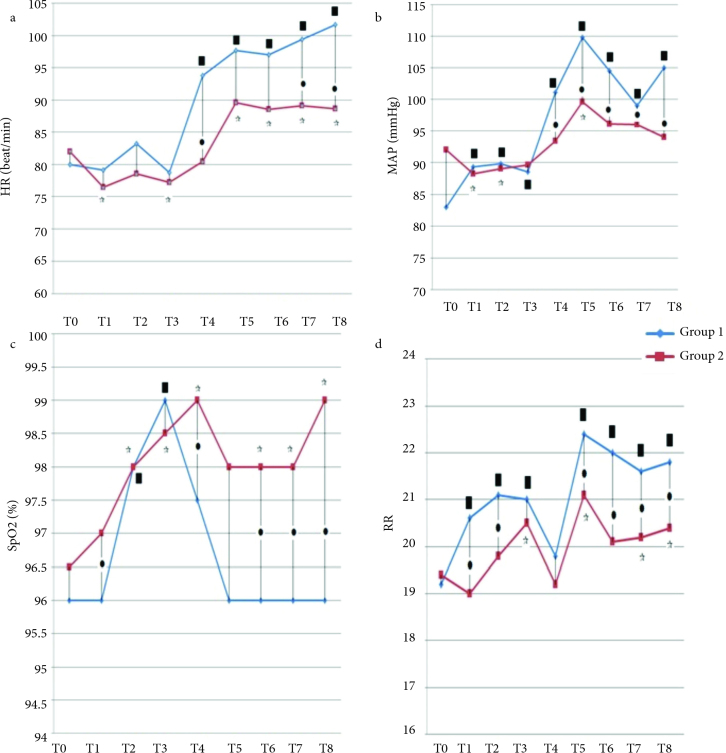
*P < 0.05. HR: Heart rate MAP = Mean arterial pressure. SpO_2_: Peripheral oxygen saturation. RR: Respiratory rate. Comparing data between the groups. P value was considered as 0.0056 after Bonferroni correction for comparison within Group 1. ☆P value was considered as 0.0056 after Bonferroni correction for comparison within Group 2.

The HR values were significantly higher in Group 1 compared to Group 2 at T4, T7, and T8 time periods (T4: P = 0.008, T7: P = 0.031, T8: P = 0.013) (Figure 2a). The MAP values were significantly higher in Group 1 at T4, T5, T6, T7, and T8 periods (T4: P = 0.04, T5: P = 0.007, T6: P = 0.006, T7: P = 0.006, T8: P = 0.004) (Figure 2b). When groups were compared in terms of SpO_2_, there was significant increase in Group 2 at T1, T4, T6, T7, and T8 periods (T1: P = 0.025, T4. P = 0.043, T6: P0 0.001, T7. P =0.003, T8; P < 0.01) (Figure 2c). The RR values were significantly lower in Group 2 at T1, T2, T5, T6, T7, and T8 time periods (T1: P = 0.001, T2: P < 0.001, T5: P = 0.010, T6: P < 0.001, T7: P = 0.006, T8: P = 0.006) (Figure 2d). 

The severity of coughing was found significantly lower in Group 2 at all time periods after insertion of EBUS (T5: P < 0.01, T6: P = 0.02, T7: P = 0.03, T8: P < 0.01) (Table 2).

**Table 2:** The evaluation of severity of coughing.

	Group 1 (n = 30)	Group 2 (n = 30)	P
Time/min	Median [Min–Max]	Median [Min–Max]	
T4	2 [0–3]	1 [0–2]	0.011*
T5	1 [0–2]	0 [0–2]	0.001*
T6	1 [0–2]	0 [0–2]	0.002*
T7	1 [0–3]	0 [0–2]	0.003*
T8	1 [0–2]	0 [0–1]	<0.001*

*All comparing data between groups were performed by Mann-Whitney U test. Data presented as median [Min–Max].

While ward VAS-A scores were comparable between the groups (P = 0.599), VAS-A scores in operating room were significantly lower in Group 2 (P < 0.001) (Table 3).

**Table 3:** Hospital ward and operating room anxiety scores.

	Group 1 (n = 30)	Group 2 (n = 30)	P
Anxiety scores (VAS-A)	Median [Min–Max]	Median [Min–Max]	
Hospital ward	45 [27–65]	48.5 [31–68]	0.102
Operating room	53.5 [33–95]	39.5 [29–55]	<0.001*

*P < 0.05: VAS-A: Visual analog scale for anxiety. Data presented as median [Min–Max].

The duration of procedure was similar in both groups (P = 0.928). However, anesthetic consumptions were significantly higher in Group 1 (P = 0.025). The number of high blood pressure and tachycardia attacks was significantly lower in Group 2 (P < 0.001, P = 0.034). Desaturation was observed in 9 patients in the placebo group and in 2 patients in the pregabalin group (P < 0.045). We observed laryngospasm only one patient in Group 1. Moreover, recovery time was significantly shorter in Group 2 (P < 0.001) (Table 4).

**Table 4:** Duration of procedure, anesthetic agent consumptions, complications, and recovery time.

	Group 1 (n = 30)	Group 2 (n = 30)	P
	Median [Min–Max]	Median [Min–Max]	
Duration of procedure (min)	17 [11–26]	17 [11–26]	0.928
Additional dose requirement(n)	2 [0–6]	2 [0–3]	0.022*
Total propofol dose (mg)	50[20–90]	40[20–60]	0.014*
Total ketamine dose (mg)	50[20–90]	40[20–60]	0.014*
Number of high blood pressure attacks ppressure attacks	4 [0–9]	0 [0–3]	<0,001*
Number of tachycardia attacks	0 [0–5]	0 [0–5]	0.033*
Desaturation	9 (%30)	2 (%6.7)	0.045*
Recovery time (min)	15 [10–25]	10 [10–15]	0.001*

*P < 0.05: between the groups. Data presented as median [Min–Max] and number/percentage.

There was no difference between the groups in terms of amnesia, acceptability of the procedure, and repeated procedures in the future (P = 0.136, P = 0.136, P = 0.237). While bronchoscopists reported good-excellent satisfaction rate as 73.3%, (n = 22) in Group 1, this rate was reported as 100%, (n = 30) in Group 2 (P < 0.001).

## 4. Discussion

In this study, we found that 150 mg oral pregabalin administration before EBUS-TBNA under ketamine-propofol sedation provided better hemodynamic responses, restricted coughing reflex, and lower anxiety scores. We also found decreased anesthetics consumption, lower complication rate, and shorter recovery time with the oral pregabalin group. The adequate comfort related to EBUS-TBNA procedure was supplied by preemptive pregabalin in addition to sedation.

Perioperative anxiety and its’ undesirable consequences such as hemodynamic disturbance is a challenge in medical practice. Anxiety causes significant emotional disturbance in diagnostic and therapeutic interventions. Moreover, anxiety is also related to high blood pressure, arrhythmia, and increased myocardial oxygen consumption [4].

Ketamine and propofol mixture is a preferred combination for sedation in interventional procedures. Ketofol provides reliable sedation condition using drug doses lower than typically required for each agent. The undesirable effects of ketamine such as nausea and cognitive disturbance are counterbalanced by the sedative and antiemetic effects of propofol [12]. Ketamine and propofol combination is known as safe and effective for sedation [13]. Respiratory depressions are one of the most serious complications during sedation. While it can happen during midazolam and propofol sedation, ketamine has lower respiratory depression incidence due to preserved airway reflexes [13,14]. The researchers applied ketofol to the patients in the emergency department, and only three patients had transient hypoxia [15]. In our study, we used ketamine-propofol combination in both groups. Since the risk of pulmonary complications could be frequent in our study population, ketofol might be a good anesthetic choice to prevent such complications. We didn’t encounter any respiratory complications that required cancellation of procedure or need for endotracheal intubation. 

Although ketofol is a satisfactory combination for sedation, preoperative anxiety is an important problem in the perioperative period. Management of anxiety in preoperative period is important to prevent unintentional hemodynamic effects [4,9]. Currently studies have focused on pregabalin or gabapentin to prevent anxiety and pain in patients who underwent surgery or diagnostic procedures. 150 mg oral pregabalin significantly prevents anxiety and elevation of HR and MAP during endotracheal intubation, compared to placebo and 75 mg pregabalin [9,16]. In a study evaluating the effect of oral pregabalin and gabapentin on hemodynamic response to laryngoscopy and intubation, it was observed that both pregabalin and gabapentin caused a significant decrease in SBP, DBP, and MAP. [16]. In the current study, hemodynamic values were significantly lower in the pregabalin 150 mg group. While hemodynamic parameters increased in both groups, increases in those parameters were significant in the placebo group. Pregabalin was effective to prevent high blood pressure and HR acceleration. In our study, SpO_2_ values were significantly higher in the 150 mg pregabalin group. Higher SpO_2_ level in the pregabalin group might be related to lower anesthetic consumptions due to preemptive effect of pregabalin. 

Studies related to the effects of pregabalin on preprocedural anxiety have been limited [17,18]. Polat et al. [18] demonstrated that preoperative pregabalin use had a positive effect on preoperative anxiety scores, and postoperative analgesia in patients had elective abdominal hysterectomy. Similarly, 150 mg pregabalin reduced the anxiety level in our study. These results showed that preprocedural pregabalin could be useful to reduce anxiety. 

Increase in airway secretions by ketamine is well-known. However, ketamine also has bronchial muscle relaxant effect and modulates the cough reflex, which is important for airway intervention such as EBUS-TBNA [19, 20]. Although there are limited data on the use of pregabalin in chronic cough [21], we have not found any data on acute cough. We administered ketamine in both study groups and observed lower coughing rate only in the 150 mg pregabalin group. This result made us think that pregabalin might be helpful to reduce coughing rate during the EBUS-TBNA procedure. The mechanism of lower coughing rate in the pregabalin group is not clear. Further studies might be required to clarify this mechanism.

General anesthesia and moderate sedation are the methods used in EBUS-TBNA procedures. However, the superiority of these methods over each other is still a controversial issue. Many issues such as the center, the availability of a suitable unit for general anesthesia, and the experience of the practitioner affect this situation [22,23]. Sedation during EBUS-TBNA provides better tolerability for patients and good conditions for operators. A comparative study showed that sedation was more reliable than general anesthesia for EBUS-TBNA, and it also provides better recovery [22]. Conscious sedation was also tolerable and safe for EBUS-TBNA procedure [2,22- 24]. In our study, the procedure was more comfortable for patients and bronchoscopists in the 150 mg pregabalin group. This result supported that the effectiveness of pregabalin was satisfactory in terms of comfort of procedure for both physicians and patients.

Studies showed that preoperative gabapentin and pregabalin reduced the requirement of opioid during postoperative period [18,25]. The previous study proved that single oral preoperative dose of 150 mg pregabalin, which was used for postthoracotomy pain control, alleviated pain scores and epidural opioid consumption in the early period[25].  In another study, it was shown that a single dose of pregabalin 150 mg reduced the requirement for intraoperative sedation and postoperative analgesic consumption. [26]. These studies were related to premedication for surgery, but we couldn’t find any studies for interventional bronchoscopy. In our study, preprocedural pregabalin administration significantly reduced the consumption of anesthetics during procedure. We also observed positive effect of lower anesthetic consumption in terms of recovery time after the procedure in the 150 mg pregabalin group.

Although various complications have been reported by the authors during EBUS-TBNA or fiber optic bronchoscopy procedure, sedation related complications rate was low [27,28]. In a study performed in 558 patients who underwent FOB, no complications were found related to sedation. In our study, we confronted with desaturation in the placebo group more frequently, and we also observed laryngospasm only one patient in placebo group. However, hemodynamic variations, desaturation and laryngospasm ended without the need to terminate the procedure in any patient. This condition showed that sedation during EBUS-TBNA can be safe with the appropriate anesthetic agents and titration. 

There are some limitations in our study. The use of end-tidal CO_2_ monitoring and bispectral index monitoring would provide additional safety. Comprehensive psychological tests could be used to measure anxiety levels, but those are not practical during an outpatient setting. VAS might be a practical and easy method to evaluate the anxiety level during the preoperative period as some researchers have done. 

We thought that preemptive oral pregabalin, in addition to sedation with ketamine-propofol combination, was effective in providing limited hemodynamic/respiratory responses, restricted coughing reflex, and lower anxiety scores during EBUS-TBNA. Besides, with pregabalin usage, decreased anesthetics consumption, lower complication rates, and shorter recovery time might have contributed to the safety of the procedure and comfort of the bronchoscopist and patients. 

## Informed consent

The study protocol received institutional review board approval (14/08/2013, ID: 366) and all participants provided informed consent.
